# Screening and validation of double allele-specific binding F-primers for the measurement of antihypertensive pharmacogenomics

**DOI:** 10.3389/fmed.2023.1269221

**Published:** 2023-12-20

**Authors:** Yang Ping, Su Quanlin, Hu Yue, Zhang Jing, Lan Wenjun

**Affiliations:** Institute of Biomedical Engineering, Qilu University of Technology (Shandong Academy of Sciences), Jinan, Shandong, China

**Keywords:** allele-specific (AS), F-primers, polymorphism, antihypertensive, genotyping

## Abstract

**Objective:**

Previous studies have proposed that genetic polymorphisms of CYP2D6^*^10, ADRB1, NPPA, CYP3A5^*^3, ACE, CYP2C9^*^3, and AGTR1 are involved in antihypertensive pharmacogenomics. The purpose of this study is to develop an amplification analysis using double allele-specific (AS) binding primers for accurate measurement of antihypertensive pharmacogenomics.

**Methods:**

To establish a quadruplex quantitative PCR (qPCR) analysis for genotyping of CYP2D6^*^10, ADRB1 (1165 G>C), NPPA (2238 T>C) and CYP3A5^*^3, and a triplex qPCR analysis for genotyping of ACE (I/D), CYP2C9^*^3 and AGTR1 (1166 A>C), mismatch AS F-primers were screened by detection of plasmid/gDNA, and were validated by agreement analysis/reproducibility evaluation, in which the ΔCq (differences in threshold cycles between the wild-type F-primer-based amplification assay and the mutant-type F-primer-based amplification assay) was employed to determine genotypes.

**Results:**

Seven pairs of primers were successfully selected through three rounds of F-primers screening. Except for ADRB1, the robustness assessment showed the amplification efficiency ranging from 0.9 to 1.1. In agreement analysis, two specimens in the training set (n = 203) were defined by the triplex analysis rather than NGS as heterozygotes for ACE, which was evidenced by gel electrophoresis. Reproducibility evaluation demonstrated that the coefficient of variation (CV) was <5%.

**Conclusion:**

Multiplex amplification analysis using screened AS binding primers is a simple, reliable, and accurate tool to guide drug delivery in antihypertensive personalized treatment.

## 1 Introduction

Hypertension is becoming the main cause of cardiovascular disease (1–4). Patients with hypertension reached 1.278 billion worldwide in 2019 (5). A previous study reported that intensive hypertension control could avoid 2.209 million coronary heart disease (CHD) events, 4.409 million stroke events, and 75,100 cardiovascular deaths in 10 years (6). However, hypertension control is still poor because of insufficient clinical experience and apparent drug resistance (2).

Clinical practices have displayed the heterogeneity of patients' responses to antihypertensive drugs (7, 8). Associated with the efficacy of antihypertensive drugs covering beta-blockers, diuretics, calcium channel blockers (CCBs), angiotensin-converting enzyme (ACE) inhibitors, and angiotensin receptor antagonists (ARBs), the hypertensive pharmacogenomics of CYP2D6^*^10, adrenergic receptor beta 1 (ADRB1, 1165 G>C), natriuretic peptide type A (NPPA, 2238 T>C), CYP3A5^*^3, ACE (I/D), CYP2C9^*^3, and angiotensin II receptor 1 (AGTR1, 1166 A>C) have been documented. For example, genetic polymorphisms of AGTR1 and cytochrome P450 oxidase (CYP2C9) impact ARB drug-target affinity and drug metabolism, respectively. ARBs exert their anti-hypertensive effect by blocking the binding of the AGTR1 receptor with angiotensin II ligands. A carrier with a C-type allele for AGTR1 appears more sensitive to ARBs (9–11). Metabolizing most ARBs, CYP2C9 is a member of the cytochrome P450 oxidase superfamily (12). Patients with the CYP2C9^*^3 (^*^1/^*^1) allele extend their fast metabolic response to ARBs (13, 14).

Discrimination of single nucleotide polymorphisms (SNPs) has become a field of intense investigation, and various technologies have been suggested (15). It is not suitable for next-generation sequencing (NGS) technology to measure SNPs due to expensive equipment and time consumption (16). Presenting high sensitivity, high throughput, and high reliability, quantitative PCR (qPCR) analysis has become a mainstream technique for SNP measurement (17–19). Uniplex amplification analysis for CYP2D6^*^10, ADRB1 (1165 G>C), NPPA (2238 T>C), CYP3A5^*^3, ACE, CYP2C9^*^3, and AGTR1 (1166A>C) has been reported (7, 20–23). In this study, we described a multiplex qPCR analysis using double allele-specific (AS) binding F-primers that included wild-type and mutated-type F-primers to discriminate above genetic polymorphisms.

## 2 Materials and methods

### 2.1 Design strategy

To detect the genotypes of CYP2D6^*^10, ADRB1 (1165 G>C), NPPA (2238 T>C), CYP3A5^*^3, ACE (I/D), CYP2C9^*^3, and AGTR1 (1166 A>C), AS F-primers with the second, third, or fifth mismatched base at the 3′-terminal, co-hydrolysis probe, and reverse primers were designed.

**Figure 1:**
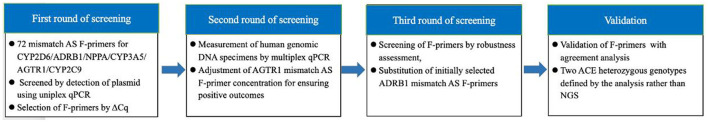
Design strategy of this study. F-primers as polymorphism-binding oligonucleotides were optimized through three rounds of screening and validated with agreement analysis considering next-generation sequencing as a reference method.

First, F-primers were screened by plasmid determination using uniplex qPCR. Second, selected F-primers were screened by detecting genomic DNA using multiplex qPCR, including a quadruplex analysis for CYP2D6^*^10, ADRB1, NPPA, and CYP3A5^*^3 and a triplex analysis for ACE (I/D), CYP2C9^*^3, and AGTR1. Each of the multiplex analyses contained two reactions: the wild-type F-primer-based amplification assay and the mutant-type F-primer-based amplification assay, in which ΔCq (differences in threshold cycles between the wild-type F-primer-based assay and the mutant-type F-primer-based assay) was employed to determine genotypes. Third, the F-primers were screened by robustness assessment. Finally, the screened F-primer-based assay was validated by concordance analysis, using NGS as the reference method. The sterilized distilled water instead of DNA was used as the negative control during the whole experiment process. The design strategy of this study is shown in Figure 1.

### 2.2 Sample collection and DNA extraction

A total of 203 oral swab samples were collected from Chinese volunteers. Genomic DNA was isolated using the QIAamp DNA Mini kit (Cat No.51304, QIAGEN, Dusseldorf, Germany), according to the manufacturer's instructions. The quality and quantity of DNA were determined by the NanoPhotometer P360 (Implen GmbH, Munich, Germany). Sanger sequencing was conducted by Personal company (Qingdao Personal Biotechnology Co., Ltd., China). NGS sequencing was conducted by the Center for Molecular Diagnosis at Shandong Provincial Hospital, affiliated with Shandong First Medical University (Jinan, China).

### 2.3 Primers and probe design

Based on the nucleotide sequences of these seven genes, AS F-primers, co-reverse primers, and hydrolysis probes were designed using Primer Express 3.0. The probes for CYP2D6^*^10, ADRB1 (1165 G>C), NPPA (2238 T>C), and CYP3A5^*^3 in the quadruplex analysis were labeled with the fluorescent dyes FAM, VIC, NED, and CY5 at their 5′ ends and the quencher BHQ1, BHQ1, BHQ2, and BHQ3 at their 3′ ends, respectively. The probes for ACE (I/D), CYP2C9^*^3, and AGTR1 (1166 A>C) in the triplex analysis were labeled with the fluorescent dyes FAM, NED, and CY5 at their 5′ ends, and the quencher BHQ1, BHQ2, and BHQ3 at their 3′ ends, respectively. Primers were synthesized by Sangon Biotech (Shanghai) Co. Ltd. (Shanghai, China), and TaqMan probes were synthesized by Invitrogen Corporation (Shanghai, China). According to NCBI, each approximately 400-bp fragment of CYP2D6^*^10 (rs1065852), ADRB1 (rs1801253), NPPA (rs5065), CYP3A5^*^3 (rs776746), ACE (rs4646994), CYP2C9^*^3 (rs1057910), and AGTR1 (rs5186) was chemically synthesized and cloned into pUC57 plasmid vectors by Sangon Biotech Co., Ltd. (Shanghai, China).

### 2.4 Multiplex AS qPCR

Optimized multiplex AS qPCR was executed in a total of 20 μL reaction mixture containing 10 μL AceQ^®^ Universal U^+^ Probe Master Mix V2 (Vazyme, Nanjing, China), 0.2 μM of each wild/mutated-type F-primer (0.4 μM for AGTR1), 0.2 μM of each reverse primer, 0.1 μM of each hydrolysis probe, and 10 ng gDNA. The uniplex amplification analysis was conducted according to the same protocol. The qPCR protocols started with a contamination digestion step for 2 min at 37°C, and a pre-denaturation step for 5 min at 95°C, followed by 45 cycles of 95°C for 10 s and 60°C for 35 s. The fluorescence signal was collected at 60°C. These amplifications were performed on the ABI7500 Real-Time PCR Instrument (Thermo Fisher Scientific Inc., MA, USA).

### 2.5 Data analysis

Data analysis and graphing were carried out using GraphPad Prism software version 9 (GraphPad Software, Inc., San Diego, CA).

## 3 Results

### 3.1 First round of F-primers screening by measurement of plasmid using uniplex qPCR

AS F-primers with the second, third, or fifth 3′-terminal mismatched base, co-hydrolysis probes, and reverse primers were designed for each SNP. Uniplex qPCR analysis and plasmid models covering homozygotes and heterozygotes were employed to screen mismatch AS forward primers (*n* = 72) to roughly enable maximization of ΔCq (differences in threshold cycles between the wild-type F-primer-based assay and the mutated-type F-primer-based assay) (Supplementary material S1). The originally selected F-primers are shown in Table 1.

**Table 1:** Primer and probe sequences.

**Analysis**	**Gene loci**	**Sequences (5^′^ → 3^′^)**	**5^′^label**	**3^′^label**
Quadruplex qPCR	CYP2D6^*^10	WF: GCTGGGCTGCACGCTAA^*^C		
		MF: GCTGGGCTGCACGCTAG^*^T		
		R: CCTCCCTCACCTGGTCGAA		
		P: ACCAGGCCCCCTGCCACTGC	FAM	BHQ1
	ADRB1 (1165 G>C)	WF1: GCAAGGCCTTCCAGG		
		MF1: AAGGCCTTCCAGC		
		R1: CGCGGCCGGTCTCC		
		P1: TGCTCTGCTGCGCGCGC	VIC	BHQ1
		WF2: CGCAAGGCCTTG^*^CAGG		
		MF2: CGCAAGGCCTTG^*^CAGC		
		R2: TGGGTCGCGTGGCG		
		P2: ACTGCTCTGCTGCGCGCGC	VIC	BHQ1
	NPPA (2238 T>C)	WF: AGATATGTCTGTGTTCTCTTTGCAGTG^*^CT		
		MF: GATATGTCTGTGTTCTCTTTGCAGTG^*^CC		
		R: GGCAACAAGATGACACAAATGC		
		P: CAGACTGCAAGAGGCTCCTGTCCCC	NED	BHQ2
	CYP3A5^*^3	WF: GTGGTCCAAACAGGGAAGAGATG^*^T		
		MF: GTGGTCCAAACAGGGAAGAGATG^*^C		
		R: CATTATGGAGAGTGGCATAGGAGAT		
		P: CATTCGTTAAGCTGGGTGGTACATACGTGG	CY5	BHQ3
Triplex qPCR	ACE (D)	WF: ACCTGCTGCCTATACAGTCACTTTTA		
	ACE (I)	MF: GCTGGGATTACAGGCGTGATAC		
	ACE (I/D)	R: GGGACGTGGCCATCACA		
	ACE (I/D)	P: CAAGGCATTCAAACCCCTACCAGATCTG	FAM	BHQ1
	CYP2C9^*^3	WF: GTGCACGAGGTCCAGAGC^*^TACA		
		MF: GTGCACGAGGTCCAGAGG^*^TACC		
		R: CGAAAACATGGAGTTGCAGTGT		
		P: TGACCTTCTCCCCACCAGCCTGC	NED	BHQ2
	AGTR1 (1166 A>C)	WF: CAGCACTTCACTACCAAATC^*^AGCA		
		MF: AGCACTTCACTACCAAATGAT^*^CC		
		R: TTCATCGAGTTTCTGACATTGTTCT		
		P: TTGCATTAGACAGATGACGGCTGCTCG	CY5	BHQ3

WF, wild-type F-primer; MF, mutated-type F-primer; R, reverse primer; P, probe.

* Mismatched base.

### 3.2 Second round of F-primers screening by detection of gDNA using multiplex qPCR

**Figure 2:**
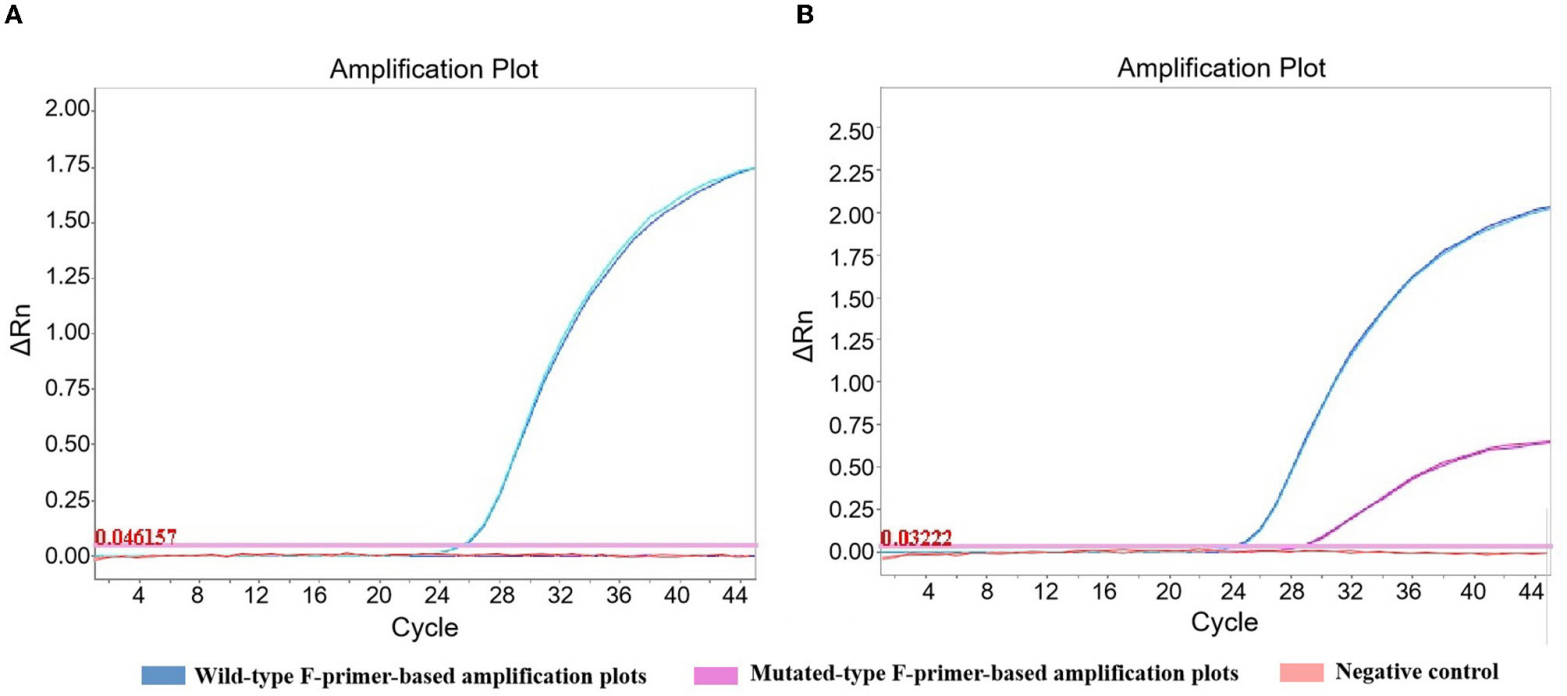
Amplification plots of AGTR1 in the triplex amplification analysis. The concentration of mutated-type F-primer for AGTR1 was adjusted from 0.2 **(A)** to 0.4 μM **(B)** to ensure positive outcomes. Wild-type homozygote was detected in duplicate by the triplex amplification analysis.

Selected F-primers were in succession screened by examination of human gDNA, comprising homozygotes and heterozygotes. To omit the positive control set in antihypertensive pharmacogenomic measurement, positive outcomes obtained from the wild- or mutated-type F-primer-based assay were required. Because undetermined results were observed in gDNA scans, the concentration of mutated-type F-primer for AGTR1 was adjusted from 0.2 to 0.4 μM to ensure positive outcomes (Figure 2).

### 3.3 Third round of F-primers screening by robustness assessment

**Figure 3:**
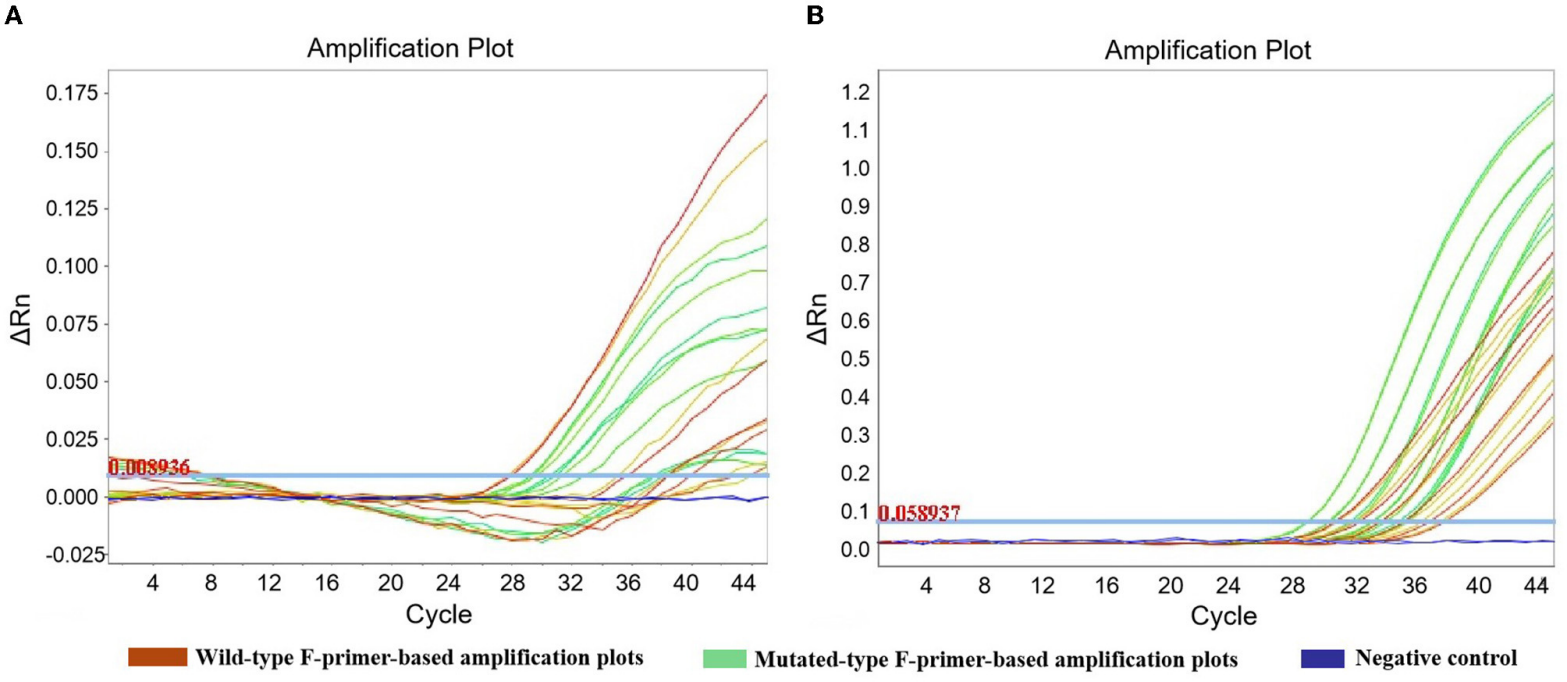
Amplification plots of ADRB1 in robustness assessment. To improve the robustness of ADRB1 measurement, F-primer WF2/MF2, probe P2, and reverse primer R2 were substituted for F-primer WF1/MF1, probe P1, and reverse primer R1, respectively, in the quadruplex amplification analysis. **(A)** Unoptimized amplification plots. **(B)** Optimized amplification plots. To assess robustness, serial dilutions of heterozygote (1.25–40 ng) were measured by the quadruplex amplification analysis, including the wild-type F-primer-based amplification assay and the mutated-type F-primer-based amplification assay. Reactions were run in duplicate.

**Figure 4:**
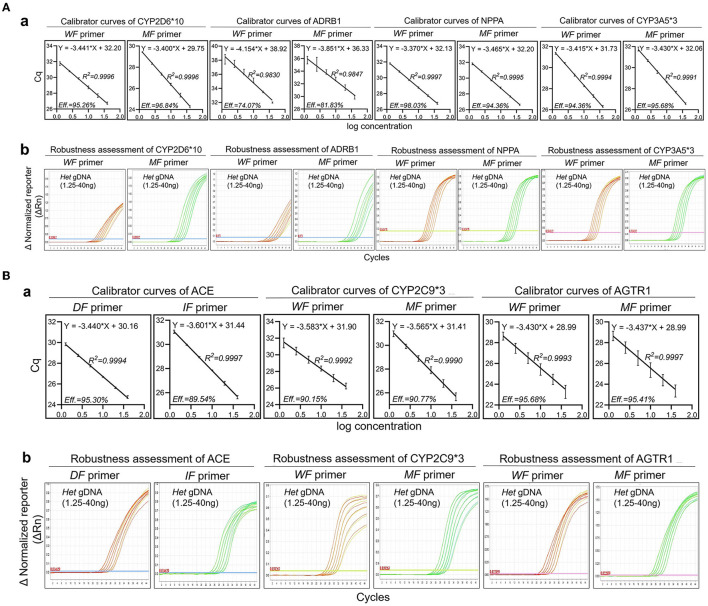
Robustness of optimized amplification analysis. The robustness assessment was executed by employing mismatch allele-specific F-primers targeting single nucleotide polymorphisms to simultaneously detect heterozygotes. Serial dilutions of heterozygotes (1.25–40 ng) were measured by multiplex amplification analysis containing a wild-type F-primer-based amplification assay and a mutated-type F-primer-based amplification assay. **(A)** (a) Calibrator curves of quadruplex amplification analysis. Amplification efficiency (Eff) % and R^2^ are shown; (b) Amplification plots of the robustness assessment in quadruplex amplification analysis. Representative amplification plots are shown. **(B)** (a) Calibrator curves of triplex amplification analysis. Amplification efficiency (Eff) % and R^2^ are shown; (b) Amplification plots of the robustness assessment in triplex amplification analysis. Representative amplification plots are shown. Reactions were run in duplicate with three independent experiments. Data are expressed as mean ± SE. *DF*, deletion-type F-primer; *IF*, insertion-type F-primer; *WF* primer, wild-type F-primer; *MF* primer, mutated-type F-primer; *Het*, heterozygote.

Six concentrations (40 ng, 20 ng, 10 ng, 5 ng, 2.5 ng, and 1.25 ng) of heterozygotes from oral swabs were prepared. Amplification efficiency was calculated using the generated calibrator curve: 10^−1/slope^−1, with the logarithm of the template copies plotted on the X-axis and Cq plotted on the Y-axis (24). The reactions were conducted in duplicate with three dependent experiments. As the calibrator curve did not appear in a dose-dependent manner, F-primer WF2/MF2, probe P2, and reverse primer R2 for ADRB1 were substituted for F-primer WF1/MF1, probe P1, and reverse primer R1, respectively, in the quadruplex amplification analysis (Table 1 and Figure 3). Except for ADRB1, optimized calibrator curves demonstrated amplification efficiencies ranging from 0.9 to 1.1 and analytical sensitivities of at least 1.25 ng (Figure 4).

### 3.4 Verification of screened F-primers-based analysis by agreement analysis

**Figure 5:**
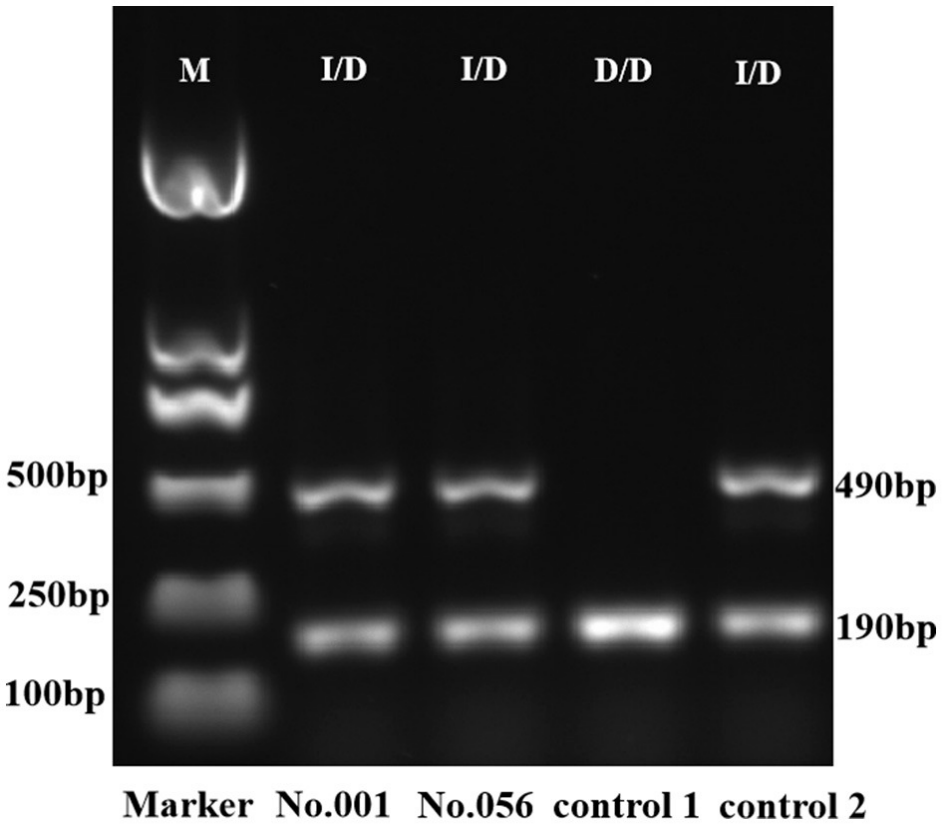
Gel electrophoresis of PCR amplicon for ACE. The D/D genotype was identified by the presence of a single 190-bp amplicon, and the I/D genotype extended both 490-bp and 190-bp amplicons. The ACE genotypes of controls 1 and 2 were D/D and I/D, respectively, defined by Sanger sequencing. M, DNA marker; I/D, heterozygote; D/D, deletion homozygote.

Considering NGS as a reference method, we examined 203 gDNA samples extracted from oral swabs to evaluate the accuracy of the multiplex analyses using double allele-specific binding F-primers. The results showed that, besides ACE, the coincidence rate was 100%. Two specimens (No.001 and No.056) in the training set (*n* = 203) were defined by the analysis rather than NGS as heterozygotes for ACE, which was evidenced by gel electrophoresis (Figure 5). The cutoff values for genotyping are shown in Table 2.

**Table 2:** Genotyping cutoff values.

**Analysis**	**Genetic polymorphism**	**Wild-type F-primer-based assay**	**Mutated-type F-primer-based assay**	**Heterozygote type**
Quadruplex qPCR	CYP2D6^*^10	mCq > wCq and mCq - wCq > 3	wCq > mCq and wCq - mCq > 3	| wCq - mCq | ≤ 3
	NPPA (2238 T>C)			
	CYP3A5^*^3			
	ADRB1 (1165 G>C)	mCq > wCq and mCq - wCq > 4	wCq > mCq and wCq - mCq > 4	| wCq - mCq | ≤ 4
Triplex qPCR	ACE (I/D)^a^	mCq > wCq and mCq - wCq > 2	wCq > mCq and wCq - mCq > 2	| wCq - mCq | ≤ 2
	AGTR1 (1166 A>C)			
	CYP2C9^*^3	mCq > wCq and mCq - wCq > 1.5	wCq > mCq and wCq - mCq > 1.5	| wCq - mCq | ≤ 1.5

mCq, Cq value of the mutated-type F-primer-based assay; wCq, Cq value of the wild-type F-primer-based assay.

a Deletion type for ACE was expressed as wild type, while insertion type for ACE was expressed as mutated type.

### 3.5 Substantiation of screened F-primers-based analysis by producibility evaluation

**Figure 6:**
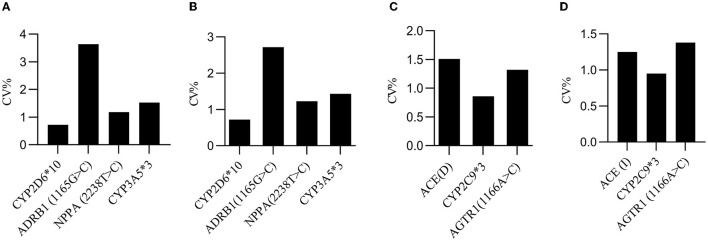
Producibility evaluation. **(A)** The inter-day CV values for the wild-type F-primers-based amplification assay in quadruplex analysis. **(B)** The inter-day CV values for the mutated-type F-primers-based amplification assay in quadruplex analysis. **(C)** The inter-day CV values for the wild-type F-primers-based amplification assay in triplex analysis. **(D)** The inter-day CV values for the mutated-type F-primers-based amplification assay in triplex analysis. The inter-day CV value was <4% for all days, specimens, replicates, operators, and reagent lots combined.

To evaluate the producibility of the analysis, each heterozygote was tested in eight-plicates by two operators, using two different reagent lots every 5 days (*n* = 80/specimen) at one site. A total of 80 Cq values were collected to calculate the coefficient of variation (CV). The results showed that the CV values for reproducibility were within 4.00% for all days, specimens, replicates, operators, and reagent lots combined (Figure 6).

## 4 Discussion

In this study, multiplex amplification analysis was established for the measurement of hypertensive pharmacogenomics. Due to genetic polymorphism, only about one-third of patients with hypertension accept effective treatment (25–30). Therefore, this study is helpful for hypertension patients to take more effective and well-tolerated medication.

Compared to other methods (31), multiplex qPCR behaves as a simple and effective approach to the detection of pharmacogenomic SNPs (32). Polymorphism-specific binding molecules in PCR-based analysis comprise dsDNA-binding dye, AS probe, and primer (33, 34). The dsDNA-binding dye-based high-resolution dissolution curve (HRM) assay needs a specific equipment module. In addition to the diseconomy, it is time-consuming and laborious to discover an appropriate minor groove binder (MGB) probe (35). The wild-type allele reaction probably outcompetes the mutated-type allele reaction when two AS probes barcoded with different fluorophores are utilized to identify genetic polymorphism (36). Sometimes, it is difficult to accurately discriminate SNPs using single-color melting curve analysis. For enhancement of AS primer specificity, base mismatch is more economical than locked nucleic acid (LNA) decoration (37). In this study, mismatch AS primers were screened and validated. The combination of a wild-type AS F-primer-based amplification assay with a mutated-type AS F-primer-based amplification assay was utilized to obtain ΔCq to define the genotype. The results evidenced that screened F-primer-based amplification analysis is a simple, accurate, and reliable approach to measure antihypertensive pharmacogenomics.

As the definition of ACE (I/D) genotype for two specimens differed between triplex analysis and NGS, we utilized PCR-gel electrophoresis to substantiate the outcomes. Located on chromosome 17, the ACE gene consists of 26 exons and appears as a polymorphism in the form of either insertion (I) or deletion (D) of a 287-bp Alu repeat sequence in intron 16. The ACE (I/D) allele can be detected by PCR using the primers flanking the 287 bp insertion sequence (38). In gel electrophoresis, the I/I genotype can be identified by the presence of a single 490 bp amplicon, the D/D genotype can be recognized by the presence of a single 190 bp product, and the I/D genotype extends both 490 and 190 bp amplicons (39). The results of gel electrophoresis validated the accuracy of the triplex analysis for ACE (I/D) measurement (Figure 5).

Based on hypertensive pharmacogenomics of CYP2C9^*^3, ADRB1(1165 G>C), AGTR1 (1166 A>C), CYP2D6^*^10, ACE (I/D), CYP3A5^*^3, and NPPA (2238 T>C), the principle of personalized drug delivery was proposed as follows: (a) doubling the standard dose is suggested when the hypertension is moderately sensitive to certain anti-hypertensive drugs; and (b) the minimum dose is recommended to initiate treatment when the hypertension is highly sensitive to certain anti-hypertensive drugs (7). Following the above principle, clinical studies evidenced that, compared to clinic experience-guided anti-hypertensive therapy, genotype-guided treatment appeared more effective and had fewer side effects. Herein, we established a simple, efficient, and accurate method for simultaneously detecting the genotypes of CYP2D6^*^10, ADRB1 (1165 G>C), NPPA (2238 T>C), CYP3A5^*^3, ACE, CYP2C9^*^3, and AGTR1 (1166 A>C) by screening and verification of mismatched AS F-primers.

## 5 Conclusion

As an accurate and reliable approach, the analysis described in this study is a valuable tool to determine the genotypes for CYP2D6^*^10, ADRB1, NPPA, CYP3A5^*^3, ACE, CYP2C9^*^3, and AGTR1, which can guide drug delivery in antihypertensive treatment to ensure curative effect. Employing the similar technique verified in this study, our laboratory will design and develop a multiplex amplification analysis for guiding aspirin delivery in the future.

## Data availability statement

The original contributions presented in the study are included in the article/Supplementary material, further inquiries can be directed to the corresponding authors.

## Ethics statement

This study was performed in accordance with the ethical standards as laid down in the 1964 Declaration of Helsinki and were approved by the Biomedical Research Ethic Committee of Shandong Provincial Hospital.

## Author contributions

YP: Data curation, Validation, Writing—original draft, Formal analysis. SQ: Writing—original draft, Data curation. HY: Software, Writing—original draft, Methodology. ZJ: Supervision, Writing—review & editing, Project administration, Methodology. LW: Funding acquisition, Project administration, Supervision, Writing—review & editing, Methodology.
