# Supplementary material for: Screening and validation of double allele-specific binding F-primers for the measurement of antihypertensive pharmacogenomics
Source: Front Med (Lausanne). 2023 Dec 20;10:1269221. doi: 10.3389/fmed.2023.1269221 (PMC10761462; doi:10.3389/fmed.2023.1269221)
Supplement: Supplementary file 1 [file Table_1.DOCX]

**Supplementary material S1** Screened results of mismatch AS F-primer

| Target | residue mismatch loci at 3' end of F-primer | Base substitution | F-primer-based assay | Specimen genotype | | |
| --- | --- | --- | --- | --- | --- | --- |
|  |  |  |  | wild-type | heterozygote | mutated-type |
| CYP2D6 | second | C>A | W | 28.04 ± 0.226 | 29.09 ± 0.127 | 31.59 ±0.212 |
|  |  |  | M | 38.60 ± 0.042 | 33.19 ± 0.262 | 31.37 ± 0.042 |
|  |  | C>G | W | 27.14 ± 0.078 | 27.56 ± 0.042 | 28.88 ± 0.290 |
|  |  |  | M | 33.42 ± 0.014 | 28.52 ± 0.071 | 26.84 ± 0.035 |
|  | third | A>C | W | 27.63 ± 0.064 | 28.04 ± 0.007 | 29.45 ± 0.007 |
|  |  |  | M | 33.71 ± 0.085 | 28.68 ± 0.078 | 26.87 ± 0.021 |
|  |  | A>G | W | 28.48 ± 0.000 | 28.55 ± 0.028 | 29.13 ± 0.177 |
|  |  |  | M | 33.28 ± 0.156 | 28.79 ± 0.042 | 26.65 ± 0.064 |
|  | fifth | C>A | W | 26.63 ± 0.028 | 26.91 ± 0.042 | 28.22 ± 0.028 |
|  |  |  | M | 29.85 ± 0.304 | 27.48 ± 0.092 | 25.72 ± 0.078 |
|  |  | C>G | W | 27.04 ± 0.028 | 27.28 ± 0.014 | 28.83 ± 0.106 |
|  |  |  | M | 29.69 ± 0.071 | 27.75 ± 0.064 | 26.08 ± 0.099 |
| ADRB1 | second | G>C | W | undetermined | undetermined | undetermined |
|  |  |  | M | undetermined/31.27 | 30.92 ± 0.283 | Undetermined/31.26 |
|  |  | G>T | W | 30.75 ± 0.078 | Undetermined/31.74 | undetermined |
|  |  |  | M | undetermined | 31.64 ± 0.149 | 30.89 ± 0.198 |
|  | third | A>T | W | 33.07 ± 0.240 | 33.87 ± 0.127 | undetermined |
|  |  |  | M | undetermined | 33.15 ± 0.233 | 32.36 ± 0.078 |
|  |  | A>G | W | 28.49 ± 0.035 | 29.23 ± 0.325 | Undetermined/29.5 |
|  |  |  | M | undetermined | 31.32 ± 0.382 | 30.19 ± 0.255 |
|  | fifth | C>A | W | 32.33 ±0.042 | 34.00 ± 0.050 | undetermined |
|  |  |  | M | undetermined | 32.75 ± 0.304 | 31.69± 0.021 |
|  |  | C>G | W | 34.26 ± 0.120 | 35.68 ± 0.014 | undetermined |
|  |  |  | M | undetermined | 33.44 ± 0.177 | 32.71 ± 0.028 |
| NPPA | second | C>T | W | 29.71 ± 0.007 | 31.07 ± 0.191 | 37.82 ± 0.969 |
|  |  |  | M | 37.67 ± 0.332 | 30.66 ± 0.127 | 30.04 ± 0.177 |
|  |  | C>G | W | 29.72 ± 0.424 | 30.67 ± 0.891 | 41.92 ± 0.127 |
|  |  |  | M | 39.49 ± 2.136 | 30.76 ± 0.163 | 30.20 ± 0.021 |
|  | third | A>C | W | 30.24 ± 0.417 | 31.34 ± 0.064 | 36.04 ± 0.269 |
|  |  |  | M | 37.52 ± 0.127 | 30.73 ± 0.106 | 30.51 ± 0.163 |
|  |  | A>G | W | 30.16 ± 0.198 | 31.24 ± 0.021 | 34.28 ± 0.304 |
|  |  |  | M | 34.64 ±0.177 | 30.65 ± 0.007 | 30.24 ± 0.106 |
|  | fifth | G>T | W | 29.83 ± 0.530 | 30.88 ± 0.134 | 32.52 ± 0.219 |
|  |  |  | M | 34.52 ± 0.035 | 30.80 ± 0.127 | 30.23 ± 0.382 |
|  |  | G>C | W | 30.08 ± 0.219 | 31.36 ± 0.240 | 36.00 ±0.417 |
|  |  |  | M | 36.09 ± 0.134 | 30.69 ± 0.028 | 30.29 ± 0.240 |
| CYP3A5 | second | A>C | W | 31.10 ± 0.212 | 31.36 ± 0.382 | 31.15 ± 0.113 |
|  |  |  | M | 39.85 ± 0.113 | 31.71 ± 0.106 | 30.98 ± 0.042 |
|  |  | A>G | W | 31.21 ± 0.177 | 32.62 ± 0.304 | 38.20 ± 0.226 |
|  |  |  | M | 39.20 ± 0.629 | 32.07 ± 0.212 | 30.95 ± 0.509 |
|  | third | T>C | W | 31.19 ± 0.191 | 32.32 ± 0.035 | 37.81 ± 0.134 |
|  |  |  | M | 39.42 ± 0.198 | 31.72 ± 0.042 | 31.23 ± 0.290 |
|  |  | T>G | W | 31.27 ± 0.078 | 32.30 ± 0.389 | 34.52 ± 0.233 |
|  |  |  | M | 41.89 ± 0.078 | 31.86 ± 0.134 | 31.16 ± 0.071 |
|  | fifth | G>T | W | 31.20 ± 0.325 | 32.28 ± 0.318 | 36.56 ± 0.644 |
|  |  |  | M | 37.29 ± 0.035 | 31.54 ± 0.347 | 30.97 ± 0.113 |
|  |  | G>C | W | 31.49 ± 0.021 | 32.69 ± 0.007 | 37.22 ± 0.545 |
|  |  |  | M | 41.36 ± 0.071 | 31.86 ±0.177 | 31.79 ± 0.389 |
| AGTR1 | second | C>T | W | 26.58 ± 0.042 | 27.29 ± 0.028 | undetermined |
|  |  |  | M | 33.85 ± 0.594 | 26.2 ± 0.028 | 25.32 ± 0.021 |
|  |  | C>G | W | 26.46±0.057 | 27.53±0.028 | undetermined |
|  |  |  | M | 34.82 ± 0.94 | 26.29 ± 0.078 | 25.36 ± 0.014 |
|  | third | G>T | W | 26.1 ± 0.269 | 27.29 ± 0.127 | 43.46 ± 0.969 |
|  |  |  | M | 31.99 ± 0.134 | 26.24 ± 0.007 | 24.97 ± 0.396 |
|  |  | G>C | W | 26.49 ± 0.078 | 27.68 ± 0.325 | 41.39 ± 0.933 |
|  |  |  | M | 33.4 ± 0.134 | 25.93 ± 0.078 | 25.38 ± 0.042 |
|  | fifth | G>T | W | 25.29 ± 0.693 | 27.42 ± 0.156 | 42.3 ± 0.622 |
|  |  |  | M | 31.37 ± 0.552 | 26.13 ± 0.17 | 25.42 |
|  |  | G>C | W | 26.46 ± 0.092 | 27.33 ± 0.488 | 37.31 ± 1.994 |
|  |  |  | M | 32.89 ± 0.559 | 26.01 ± 0.311 | 25.51 ± 0.442 |
| CYP2C9 | second | C>T | W | 27.74 ± 0.035 | 28.75 ± 0.134 | 40.05 ± 0.354 |
|  |  |  | M | 41.45 ± 0.41 | 29.15 ± 0.163 | 28.52 ± 0.014 |
|  |  | C>G | W | 27.64 ± 0.141 | 28.61 ± 0.035 | 36.61 ± 1.223 |
|  |  |  | M | 39.96 ± 0.021 | 28.74 ± 0.064 | 28.19 |
|  | third | A>C | W | 27.85 ± 0.113 | 29.05 ± 0.071 | undetermined |
|  |  |  | M | 35.88 ± 0.148 | 29.19 ± 0.255 | 28.45 ± 0.113 |
|  |  | A>G | W | 28.12 ± 0.007 | 29.51 ± 0.021 | 40.7 ± 1.838 |
|  |  |  | M | 35.07 ± 0.354 | 29.36 ± 0.042 | 28.75 ± 0.035 |
|  | fifth | A>C | W | 28.33 ± 0.007 | 29.7 ± 0.255 | 35.36 ± 0.537 |
|  |  |  | M | 34.57 ± 0.163 | 29.29 ± 0.064 | 28.76 ± 0.042 |
|  |  | A>G | W | 28.1 ± 0.064 | 29.49 ± 0.064 | 38.29 ± 1.57 |
|  |  |  | M | 34.91 ± 0.2333 | 29.29 ± 0.05 | 28.65 ± 0.042 |

**Note**: W: wild-type. M: mutated-type. Yellow: selected wild-type F-primer. Green: selected mutated-type F-primer.
